# The role of sensous flow and sensing the ground in movement skill experiences—a reflection using the practice of yoga as an example

**DOI:** 10.3389/fspor.2023.1217607

**Published:** 2023-09-07

**Authors:** Gunn Helene Engelsrud

**Affiliations:** Institute for Sport, Nutrition and Natural Sciences, Western Norway University of Applied Sciences, Campus Sogndal, Norway

**Keywords:** phenomenology, passivity, letting be, yoga, bodily resonance

## Abstract

This contribution takes a phenomenological approach to explore the sensuous flow and perceived experiences in practicing movement skills, using the practice of yoga as a case study. The article focuses on the role of perception and the anonymous aspect of the body's responses in practicing skills and capabilities to move, with yoga as an example. The author uses a phenomenological framework, highlighting how passivity and sensuous flow is available in the practice of yoga. Edmund Husserl's concepts of passive synthesis and Thomas Fuch & Sabine Koch interpretation of bodily resonance and Kym Maclaren's “letting be” are used as analytic frames to illuminate how movement experiences are dependent on bodily awareness towards the ground, without demanding conscious willpower or focus on force, but listening and sensing with and from the body. The article aims to illuminate the ambiguous character of how movement experienced from a first-person perspective gains importance by understanding oneself, others, and the world as reciprocal and intertwined phenomena.

## Introduction

“*Embodiment as a paradigm or methodological orientation requires that the body be understood at the existential ground of culture—not as an object that is “good to think”, but as a subject that is “necessary to be”* (1)*.*

It has been suggested that the experiences of yoga practitioners, mindfulness practitioners and practitioners of Body-Mind Centering (BMC) of Feldenkrais have somatic foundations (2–4). This means a focus on the “not yet conscious” (5), but ever so sensuously flowing whole bodied experiences where the cells, nervous system, organs, etc., are places where weight, flow, space and so on constitute what these branches of research examine. To elaborate further on the experiences of somatic foundation, I have chosen to build on phenomenology, where the understanding of human movement is based on a bodily experiential and existential foundation (1, 4, 6–12).

The article is written with the aim of theorizing the phenomenon of sensuous flow in movement, with the practice of yoga as an example. The article elucidates how sensuous flow is experienced in a movement practice such as yoga. The structure of this article is an invitation to theorize the intrinsic, dialectical, and ambiguous ways that practising yoga is a way of moving oneself based on experienced of ground, touch and sensuous flow, which can be defined as somatic modes of attention. My research interest lies in examining how passivity and sensuous flow is practised with the aim of discovering quality, harmony, and rhythm, as well as boundaries and tensions, while moving. This involves examining practices as they are lived and trusting that being in movement has equal value to doing movement. Further that self-knowledge through awareness of the self, body and the world are a basis for movement experiences I have chosen elements from the practice of yoga as a case study. To set the tone of what I want to illuminate in the article, I start by re-telling (13) a situation that illuminates how bodies’ uptake of vibrations from the ground directs their movements. I find this situation relevant to use as an entrance to the moving happens from relating to the ground.

*During the 2004 tsunami, the elephants noticed that the soil and substrate were different than usual, even before the wave reached the shore. They sensed the earth through their feet and saved themselves by moving away from the sea. People, conversely, were keen to see what kind of wave was approaching the shore. The elephants extracted their bodily responses from the world they sensed and “evaluated” the situation far better than the humans, who let their concern about seeing what was happening have an impact on their actions*.

This example provides insight into the non-conceptual, yet ever so clear, understanding of the elephants' embodied connection to their surrounding environment. The elephants' actions showed how they perceived and responded to the vibrations in the earth through their feet and their whole bodies. The elephants “listening” to the processes of the “inner” body in relation to their environment and and “asking” the earth if they were safe or in danger. According to Fuchs and Koch (9), the example shows what animals do when they, through their bodily experience, examine whether a tree is climbable or whether water is drinkable, for example. So, the question is: why were the people keen to see the wave, and why did they prioritize their visual senses over kinesthetic listening, which means to listen through the skin and feel the contact with the ground, and linking this to their relationship with the earth and the ground through their feet? There can be many explanations, which I do not follow up here. What I draw attention to is the the principle that human beings embody the same relationship with the earth as animals, but as mentioned by Berg Eriksen (14), senses like vision and listening team up with intellect to create a response, while kinesthetic feeling and sensing team up with the body to do so^1^. However, as Berg Eriksen points out, a hierarchy exists between the senses, where vision affects distance and judgment and invites the subject to obtain a third person and objective point of view—while the more bodily senses such as touch, smell and taste take a first-person, subjective, and intimate point of view that requires proximity. Phenomenologically speaking, Fuchs (15 p. 222) wrote:

“We feel something ‘in the air’, or we sense an interpersonal ‘climate’, for example, a serene, a solemn, or a threatening atmosphere. Feelings befall us; they emerge from situations, persons, and objects which have their expressive qualities and which attract or repel us. This emotional space is essentially felt through the medium of the body which widens, tightens, weakens, trembles, shakes, etc. in correspondence to the feelings and atmospheres that we experience”

This means that something that might be felt as if it were “in the air” is assumed to be available as a noticeable condition in the body and that this should not be dismissed, but rather pursued. Accordingly, it follows that one's relationship to oneself, others and the world is felt and anchored in a personal relationship that in social situations, before a word is even said, we experience each other through being spoken to or ignored. Unpacking experiences without words shows us exactly how experiencing sensuous feelings take place in shared spaces and that the self, world and others belong together and “reciprocally illuminate one another and can only be understood in their interconnection” (16). In this interconnection, there are moments of responsivity which are “passive”. However, “passive” does not mean static or inactive, rather it indicates the synthetic work done by the consciousness that *goes unnoticed* while it is engaged in direct (i.e., active) experiences, which Husserl elaborated on in his paper on passive and active synthesis (17). For Merleau-Ponty, the anonymous refers to a level in perception which concerns the depth of perceptual presence (18). When the elephants sensed a vibration in the earth, they illuminated the reciprocity between sensing the earth through touching the ground and sensing the feet and body weight contact with the earth, e.g., moving and being moved. As pointed to by Bainbridge Cohen (2), yielding to the ground also creates access to moving in and through space, both to moving forward as the elephants did and also to the sensual depth of the experience and situation. It is significant and relevant to elaborate on being in *the present movement* in movement, while also being attentive to space, including to human beings moving. Such an elaboration also functions to illuminate the question that remains: why were people keen to see the wave? What kind of position did people take when they seem to prioritize watching the wave and perceiving it as something exciting through their vision? As de Jaegher (19) writes, “Characterizing knowing as a relationship of *letting be,* provides a nuanced way to deal with the tensions between the knower's being and the being of the known, as they meet in the process of knowing-and-being-known”. But what does it mean to deal with oneself as a knower of movement and what is being known? (20–23) To follow up that question, I will position the perspective of the article more clearly in phenomenology.

## Bodily experiences from a phenomenological perspective

Phenomenology is a European philosophical tradition that has had significant worldwide influence on thinking, research, architecture, art, culture, and other branches of the humanities (24). While there is no single answer to the question of what phenomenology is, it is generally agreed that Edmund Husserl (1859–1938) is the “father” of phenomenology. Husserl radicalized the theory of knowledge of his time and aimed to show that the building of knowledge based on a separation between the world and the knowing subject was rooted in an erroneous view of understanding. He wanted to broaden insight into human experience in the *lifeworld* in which people live together with one another and engage in communicative relations (24, 25). The lifeworld is a perspective that forms an existential precondition for engaging in different social worlds in which human needs and intentions are related to and in transition from one generation to another. It enables intersubjective communities and shared meanings as well as interpersonal relations and embodied affects to circulate between humans (26). Conducting research from a lifeworld perspective means understanding human beings as being inseparable in a shared world (24). However, living in a shared world also means that humans tend to be largely unaware of their own bodily movements, own language, and habits in everyday life (6). I take this as an opportunity to unfold movement experiences that we can be aware of, and benefit from. The philosopher Maurice Merleau-Ponty (1908–1961), known as the “philosopher of the body,” developed the concept of the *lived body*, in contrast to an abstract and mechanical body. He wrote:

We have relearned to feel our body: we have found underneath the objective and detached knowledge of the body that the other knowledge which we have of it in virtue of its always being with us and of the fact that we are our body. In the same way, we shall need to reawaken our experience of the world as it appears to us insofar as we are in the world through the body, and insofar as we perceive the world with our body (11).

By re-establishing the contact between the body and the world, Merleau-Ponty viewed the body as ambiguous and unfolded in a double sense (e.g., subject and object, seeing and seen, touching, and touched, sensible and sensed). In his unfinished work *Le Visible et l'invisible* (1968), he conceptualized the body as the *flesh of the world*: as both expressive and anonymous. The subject herself cannot, in certain aspects, know herself. She can say “I want to go for a walk” but not “I want my heart to beat.” Thus, the body is both present for a person and distant and alien (27), as there are phenomena in the body that live “their own life” (e.g., heartbeat or breathing). This means that when phenomenological researchers pay attention to the passivity of and sensuous flow in the body, they contribute, according to Merleau-Ponty (11), to create insight into experience of shared situations and how movement comes from- and are performed in relation to the ground, others and in and from one's own body.

Back to the the elephants, who moved themselves to safety, while human beings were curious to see and gaze at the wave. Touching the ground is an immediate sensation felt in the body. The gaze creates distance and judgment and the somatic modes of attention (1, 28). The wave probably appeared to the people as “important” or, in a strange way, “attractive” (9). This brings us to the understanding of bodily resonances underlying micro-sensations; feelings of warmth or coldness, tickling or shivering, pain, tension or relaxation, constriction, or expansion, sinking, tumbling, or lifting, etc. They correspond, on the one hand, to autonomic nervous activity (e.g., raised heartbeat, accelerated breathing, sweating, trembling, visceral reactions), and to various muscular activations, bodily postures, movements, and related kinesthetic feelings (e.g., clenching one's fist or one's jaws, moving backwards or forwards, bending over or straightening oneself, etc.) on the other. Without these sensations or others as part of our experiential knowledge, the world lacks meaning, and as pointed to by Fuchs & Koch, when people are affected by affordances of a situation, it triggers a specific bodily resonance (“affection”) which in turn influences the emotional perception and evaluation of the situation and implies a corresponding readiness to act and speak, through their energy, tone, and manner (2). From a phenomenological perspective, bodily and affective resonance and interaction with others are what allow people to understand each other.

In summary, the subjectivity and intersubjectivity in the understanding of movement experience the elephants' sensuous, and bodily response to- and the relationship with the earth that this gave them, helped them to take the decision to move in the right direction, away from danger. Far from being barriers to making correct decisions, bodily sensations relate to the knowing body and can help us to make decisions in all sorts of situations. Relying on sensation in knowledge creation is also advocated for by Hanne de Jaehger and Kym Maclaren. By including Maclaren's concept of “letting be” with regard to practicing (movement), it is possible to further elaborate on this idea in order to illustrate that the way in which the moving subject approaches and orients themselves from their experiences is where the qualities of the movement are discovered and differentiated (28). Edmund Husserl's concepts of passive synthesis and hyletic flow (19), Kym MacLarens concept of “letting be” are used as analytic frames to illuminate how movement acquisition is dependent on silence and awareness, without focus on force, but of “letting be”. From a phenomenological perspective this allows for the exploration of the body's unique capacities to interact with the ground, space and others (human and other species) as the environments they inhabit and move and create movements within [cf (30)].

## Creation of method and material through practicing yoga

As already stated, I use examples from yoga practice to help to theorize the somatic ground, bodily resonances, and value of “letting be” as part of movement acquisition that I am interested in. These examples are created from autoethnography, which is a collective term for ways to develop knowledge by creating a material by combining personal reflections in combination with reflections on cultural and institutional conditions (31, 32). My examples have been created by narrating experiences from practising yoga. In this article, I have chosen to highlight experiences from three situations. I have asked myself what some self-perceived bodily conditions in yoga practice can provide knowledge about beyond what is known and discovered about yoga practice and experience of sensuous flow. My use of this example is not intended to be interpreted as empirical fact or as experiences that are unique to me or others (6, 8, 33), but rather as actual and possible examples of “the same kind of phenomenon” (6). In line with Behnke, my interest “is eidetic, not empirical, and the specific examples chosen are merely meant as clues toward structures that could equally well be illustrated by different examples” (p. 185). It mean that when I have analyse the experiences from yoga, I have aimed at pointing to a more common humans experience of moving, that indicate how sensuous flow and passivity in receiving—and relating to the ground are shared phenomenon in experiencing movement.

## The yogi's experience of moving themselves into passivity and stillness

Yoga traditions are numerous and are of great interest to philosophers, researchers, and practitioners world-wide. Here, I touch on some situations that many practitioners have in common, a position called s*havasana*, a Sanskrit name derived from two words: *sava* meaning “corpse” and *asana* meaning “posture.” Traditionally, the *corpse* pose is an asana performed at the end of a yoga session, in which practitioners lie flat on their backs with their heels spread as wide as the yoga mat and their arms a few inches away from their body, palms facing upwards. *Shavasana* is considered a practice where the yogi gives their body to the ground and relaxes by being attentive towards breath and weight and allowing kinesthetic and somatic modes of attention that arise from the unity of the body and the spatiotemporal field. When engaging in *shavasana,* the traditional idea is that intentionality is directed towards preparing the body for death. This is a text that I, as a yoga practitioner, wrote after a yoga class:

*I am lying on my back, flat on the floor. The room is warm, and I feel the bright light even though my eyes are closed. The teacher tells us that this is our reward after the challenging session and all we have to do is relax and let our bodies sink into the floor. The floor is trusting and safe. I feel my body tensing up in the shoulders—why? I feel the floor touching my body and my body touching the floor. In the beginning, the two are different, then they merge, and I am one with the floor and the space, no borders but indulging in deep relaxation. The outbreath and inbreath, just follow and are at one with my breath. Suddenly, I hear the teacher's voice, it seems to come from far away. “Take a deep breath in, a deep breath out, slowly in your own time, come back to your awareness of your body. When you are ready, take a deep stretch and roll over to your right side. Take your time to come up to a seated position”*.^4^

The experience in *shavasana* felt like being carried away from the actual space. The teacher's voice combined with listening to what was happening in my own body, the movement experience and the passive receptivity contrasted strongly with the movement experiences that preceded *shavasana* in the yoga session. Being in *shavasana* creates opportunities for someone to explore the subtle layers of being, the invisible levels, where passive synthesis and the kinesthetic mode of attention [cf (34)], accordingly, move the body into stillness. In this stillness, a type of transformation occurs, and a feeling of being one with one's circumstances arises. The yogi gives themselves over to the (spiritual) world and transforms their world, like the painter transforms the world with their painting. During the transformative phase, the yogi's attention descends to subconscious, unconscious levels which opens the way for Husserl's passive synthesis as a particularly important dimension of experience. *Shavasana* brings the yogi into subtle modes or regions of experience and cultivates a sensibility appropriate to the phenomenon of relaxation. In *shavasana*, the voluminous body often no longer feels its borders with space but is instead one with its circumstances. Nothing is actively moving, and the silence of deep relaxation floods the body. When the teacher in this situation asked the yogis to “bring awareness back to the body”, they built on their knowledge of the phenomenon of long-time practice within the tradition, and they were teaching from an experience that echoes the “understanding in [their] bones” (35). The visual process of *seeing* is no longer given a prominent position. However, *sensing* and *feeling* are the bodily functions that embrace the current moment and turn the moment into a healing one, where the sensory intake and bodily awareness towards oneself, others and the world and the other plays a significant role.

## The yogi's experience of being suspended between ground and space

*I am standing on my feet, gazing forward into space. The room is warm, and the ground feels, down, deep down, as if roots are growing. I clearly feel my feet touching the ground, I perceive the ground. I feel the surface of the ground and my feet touching. Above me is the “air” and I reach up by floating my arms above my head, touching each other—coming together. Suspended in space, grounded. As I breathe out, I let my arm follow the front of my spine, trying to meet the ground. The teacher tells me to enjoy the forward bend. For a moment, the tensile forces that held me up melt into the ground and give the weight to the ground, still not collapsing. My hands and feet together—for a moment both seeking support from the ground, have the same function: feet as hands, hands as feet*.

The base of the movement in the part of the body that touches the ground is the anchor of one's support and is an expression of the ontological understanding of the bodily movement as *a being in the world*. The double sensation of touching and feeling the gravity that I am yielding to but also pushing against in a yield and push pattern, which creates a productive tension and suspension of energy to move with, to manifest and involve relationships, and to create experiences that fluctuate in terms of the movement, meaning, and environment and ground. Such experiences are not measurable; they are part of the existential condition of living as a bodily subject in a double dialectic as both the one who touches and the one who is touched (11). The moving subject is the one who is the self-moved mover, that rests upon a positive appeal to the experienced unity of the freedom in the spatiotemporal field, and which, according to Todes (36), is a field that is produced by the way the body's specific structure both constrains and enables one's movement skills. Fuchs (10) claims that, through bodily resonance, we notice and gain an intuitive understanding of the feelings of others, and that this arises in our bodily engagement with them and the world (I refer to the elephants that noticed a new vibration in the soil). This is a perspective in which the mover clearly notices bodily somatic sensations, from which the body is mobilized in wonder and explorations of moving and being moved, like how Fuchs and Koch (9), describe what animals do when they examine whether a tree is climbable or whether water is drinkable, the mover asks the movement: can I move here, can I connect there, how do I create space and move in space?

### The primordial and non-conceptual understanding in silence

The primordial and non-conceptual understanding that yogis' experience in *shavasana* is clear to them when they compare the different movement experiences during practice. The different objects for reflection are intertwined in the relationship between being and having a body, as shown in Merleau-Ponty's example of the two hands touching. Merleau-Ponty gives credit to the primordial and silent language of the body. “Les voix du silence” refers to those who do not speak about the world, but instead let the world come to light and discover the experiences that hide behind discursive language (e.g., 37). I compare the way the painter transforms their surroundings in their painting with how *shavasana* is an art of relaxation that subtly shifts the parameters of the yogi's world when they release their body to the ground. However, doing nothing, receiving the ground, and giving up visible movement activity can be challenging. Finding relaxation in *shavasana* is also an achievement, a way of being in the “here-ness” that Husserl called the ground zero of orientation (7). At the same time, entering *shavasana* means experiencing a place where the perceptual field is simultaneously limited by closed eyes yet expanded by an open imagination and a floating feeling where the body and the world are in union.

## “Letting be”

According to Maclaren (38), it can be challenging at first glance, particularly in relation to teaching, to take in and accept the words “letting the other be” as they can lead to thoughts about encouraging a “passivating” attitude towards others, or, as one might express it in everyday speech, to allowing others to go their own way. Maclaren's idea of “letting the other be”, also relates to “letting oneself be”, and, as previously stated, expresses a fundamental way of being in the world, both subjectively and intersubjectively. It shows that in praticing yoga in an intersubjective environment yogies are exposed to each other. “Letting the other be” also means feeling *oneself* as present and being “here” and feeling the “me-ness” of being here. When it comes to the bodily experience, “letting be” relates to body weight, volume, structure, senses, thoughts, and feelings of the body as they are from moment to moment and experiencing “releasing our body weight” towards the ground and registering what this does to our openness and presence in dialogue with others (40).

Giving space to “letting be” becomes meaningful when the yogies understands themselves and others by engaging with and taking each other in through the body. Thus, before a word is said, human beings know quite a bit about the mood of a room and the quality of the contact with others in it. The fact that human beings can *notice* and *sense* others through the body is due to our bodies belonging to a common world (41). Maclaren (29), also writes that the person who lets others be is aware that for many people it means a struggle with one's own preconceptions of the Other. “Letting be” requires me to find my own free space within myself and my relationship with the Other. It means being able to suspend one's own habitual thinking and understand that movement experience occurs between and within human relationships in the world.

### Embodied learning through grounding the weight

No movement, whether it be of an elephant or a person, can be learned without relation to the ground. The body parts that touch the ground processing the bodily weight, that further are driving forces for action and movement; without the relation and releasing the weight to the ground, a movement will die or break. The mover must feel and sense the ground and skin before it becomes a “motor” action, whether it is snow under skis, rain pelting down on the body, the other's nice smile, the squealing of tram tracks, heat in the head, or a loud whistle. Allowing time *to be* is a theme explored in practices like Feldenkrais, Body-Mind Centering, Dance Improvisation, in addition to yoga. Hanne de Jaegher comments on this as follows: “Characterizing knowing as a relationship of *letting be* provides a nuanced way to deal with the tensions between the knower's being and the being of the known, as they meet in the process of knowing-and-being-known” (de Jaegher, 2019, p 1). Including the idea of “letting be” provides a perspective for exploring and reflecting upon what considering this phrase might provide in using the sensations from one's own body to help to avoid objectifying and quantifying one's movements only as products. By opening the spatiotemporal field for shared knowledge and dialog, acquisition of movement happens “by itself”. Considering the non-conceptual bodily sensations that the elephants used to escape the tsunami, human beings may practice listening to the bodily resonances and be guided in the same way as the elephants understood that they had to escape and move away from danger. The mover might, like the animal that wonders whether the tree is climbable, ask themselves if they are available for contact, with the movements, others, space, or environment. Bodily resonances will, if given attention, function as a driving force for direction and dynamic movements. Bodily resonances and affective relationships with the ground show humans as emotional bodily beings that live together with other people and circumstances in the world (36), and that the practitioner achieves embodied self-knowledge (12). One argument for embodied self-knowledge in movement acquisition practices is concentrated upon “re-achieving a direct and primitive contact with the world and endowing that contact with philosophical status. It is in the search for a philosophy which shall be a ‘rigorous science’, but it also offers an account of space, time, and the world as we ‘live’ them” (11). We can take inspiration from Merleau-Ponty's approach to acknowledge that a person's own subjective bodily experiences can count as knowledge. A subjective perspective means that the living and lived body, from a first-person point of view, is not a perspective that could be observed from other perspectives: “It is our very manner or way of being in the world and, as such, it allows us to adopt perspectives in the world. Thus, we “are” our bodies in a fundamental sense” (41). I take this as an argument to include bodily self-knowledge, which means discussing the role of experiential knowledge, subjective knowledge, embodied knowledge, self-esteem, and self-awareness in movement practice and regard those phenomena as a precondition for such knowledge in a broad sense.

## Summary—the potential for experiencing moving and being moved

I have provided examples in in favor of including sensitizing oneself to the practice of “letting be” in movement experience and include passivity, sensuous flow and the not-yet known as potentials for understanding one's own movement experiences and orientation. By highlighting emotions as driving forces for movement and action, I have tried to give space for a phenomenological, existential, and relational ground for practicing movement with inspiration from the elephants and situations from yoga practice. To learn about oneself by noticing the qualities of one's own body, spontaneity, or lack thereof, experiencing of what is tense, loose, available, or stuck is to discover and gain insight into the way one moves and are moved. The double sensation of moving and being moved, touching, and being touched give a clear insight into the orientation towards how movement is experienced in yoga practice. By highlighting that human bodily emotion and sensation are a basic approach to movement and a basis for knowledge, the concepts of bodily resonances and “letting be” lay the theoretical groundwork for using interactive processes that occur in the bodily resonances, spaces, and awareness practiced by the moving subject when they use little effort and increased sensibility. Taking such perspectives into moving allows the mover to understand movement as a process that underlies any product-oriented perspective on movement. The perspective in this article supports exploration of movement and includes the “whole body as being and being moved” (9). It means letting oneself be moved by the movement in reciprocity, to act and direct one's movement to fulfill a purpose or desire. I want to make angels in the snow, lay down and rest, or take my dog for a walk. The mover's responses to oneself, others, and the world and environment are keys to understanding the role of the sensuous and passive aspects of moving and being in the world as embodied subjects, and how phenomena of “letting be” adds quality to movement experience that might also be transferred to theorizing movement experience and acquisition within other branches of research within sport sciences.

## Data Availability

The datasets presented in this article are not readily available because: the autoethnographic materials used in this article have the function of examples for highlighting the author's understanding of the movement experiences.

## Footnotes

^1^This can be viewed as a dualistic position, and I am not sure that such a split is generally valid, but it illustrates the situation with the elephants.

^4^This example is also used in (8).
